# Multi-Strategy Learning Boosted Colony Predation Algorithm for Photovoltaic Model Parameter Identification

**DOI:** 10.3390/s22218281

**Published:** 2022-10-28

**Authors:** Mingjing Wang, Long Chen, Huiling Chen

**Affiliations:** 1 School of Computer Science and Engineering, Southeast University, Nanjing 211189, China; 2 The Key Laboratory of Computer Network and Information Integration (Southeast University), Ministry of Education, Nanjing 211189, China; 3 College of Computer Science and Artificial Intelligence, Wenzhou University, Wenzhou 325035, China

**Keywords:** photovoltaic models, parameter extraction, colony predation algorithm, multi-strategy learning, level-based learning

## Abstract

Modeling solar systems necessitates the effective identification of unknown and variable photovoltaic parameters. To efficiently convert solar energy into electricity, these parameters must be precise. The research introduces the multi-strategy learning boosted colony predation algorithm (MLCPA) for optimizing photovoltaic parameters and boosting the efficiency of solar power conversion. In MLCPA, opposition-based learning can be used to investigate each individual’s opposing position, thereby accelerating convergence and preserving population diversity. Level-based learning categorizes individuals according to their fitness levels and treats them differently, allowing for a more optimal balance between variation and intensity during optimization. On a variety of benchmark functions, the MLCPA’s performance is compared to the performance of the best algorithms currently in use. On a variety of benchmark functions, the MLCPA’s performance is compared to that of existing methods. MLCPA is then used to estimate the parameters of the single, double, and photovoltaic modules. Last but not least, the stability of the proposed MLCPA algorithm is evaluated on the datasheets of many manufacturers at varying temperatures and irradiance levels. Statistics have demonstrated that the MLCPA is more precise and dependable in predicting photovoltaic mode critical parameters, making it a viable tool for solar system parameter identification issues.

## 1. Introduction

The world has recognized a strong need to transition to renewable energy sources in place of formerly destructive forms of energy [1,2]. There are other problems that have arisen because of the use of these energy sources, such as climate change and energy shortages as well as political instability [3]. Solar energy is gaining increasing interest since it can be directly converted to electricity and used to generate electricity in addition to being free, infinite, and environmentally friendly without the use of nonrenewable resources [4]. As a result, solar-based energy systems are commonly used to generate electricity via huge photovoltaic (PV) modules. However, the photovoltaic facility is frequently exposed to a severe climate and frequently succumbs to rundown conditions when confronted with strong winds or a major hurricane. This suggests that the conversion efficiency of photovoltaic systems can be significantly altered. This raises the possibility that solar systems’ efficiency of conversion may drastically deteriorate.

As a result, it is vital to monitor the real-world performance of PV modules by taking current–voltage readings during operation. A mathematical model and parameter identification are usually required to model PV systems. The single diode (SDM) and double diode (DDM) models are the most popular and commonly used [5]. However, real PV model behavior is heavily reliant on parameters that are not known in advance, which can be unpredictable due to hardware maturation, failure, and unsteady operating conditions. Precise identification of the PV parameters during development may thus be necessary to assist control, enhance PV frameworks, and eventually organize applications [6].

PV parameter identification can be considered an optimization problem, as described in [6], which is very complex and has obvious multimodal optimization characteristics. The factors that distinguish proving techniques for PV parameters are divided into two categories: deterministic algorithms and metaheuristic algorithms. The Newton–Raphson methods [7], tabular methods [8], and iterative cure fitting [9] are examples of deterministic strategies and additional computational conditions, such as convexity, composability, and differentiability of the selection space, are usually required. This sort of algorithm may not be able to handle this nonlinear and multimodal complicated problem. Additionally, they are vulnerable to the features of the beginning solution, which may have an impact on their efficiency. It is important to note that metaheuristic algorithms have recently received more attention [6,10]. For a variety of reasons, metaheuristic algorithms are getting a lot of interest. Algorithms like this one can solve problems in a black box without relying on task specifications or incidental data limits such as convexity or the distinctability of different option spaces [11,12]. Stochasticity allows this algorithm to conduct high probability leaps out of the stalemate, unlike deterministic techniques [13,14]. If a sufficient balance between exploration and exploitation can be ensured, they can also achieve high quality arrangements with a reasonably rapid and effective search [15]. Because these metaheuristic algorithms have the potential to be applied in a variety of domains such as machine learning [16], multi-threshold image segmentation [17], engineering design [18], and medical diagnosis [19].

Optimization is a vital step in order to reduce or maximize criteria while also satisfying the requirements or limitations of the decision-maker [20]. For global optimization problems [21,22], metaheuristic algorithms offer a great deal of potential [23,24]. Metaheuristic algorithms have been used in many scenarios, medical diagnoses [16,25,26,27,28], picture engineering [29,30,31], and engineering applications [32,33,34]. As a result, metaheuristic algorithms and their modifications have been widely used to evaluate the parameters of PV. ELPSO with particle swarm optimization was invented by Jordehi et al. [35]. The RTC France silicon cell, STM6-40/36 monocrystalline module, and PVM 752 GaAs thin film cell are all examples of this working successfully in solar systems. Classified perturbation mutation particle swarm optimization (CPMPSO) was used to identify alternative PV model parameters by Liang et al. [36]. A time-varying acceleration coefficient PSO (TVACPSO) was developed by Jordehi et al. [37] for this estimate problem, and the TVACPSO might outperform other rivals. Askarzadeh et al. [38] used artificial bee colony (ABC) factors to identify evidence of solar cell models. For the parameter extraction of a solar cell, genetic algorithms (GAs) were also given, and a conclusion was reached that GAs can handle this task better [39]. To properly identify solar PV module parameters, a penalty-based different evolution algorithm [40] was developed. Experiments have shown that the suggested computation is capable of extracting the parameters of solar-powered cell models using an adaptive version of DE [41]. For parameter estimation of solar photovoltaic models, additionally, Gao and his colleagues [42] utilized a parallel co-evolutionary DE [43] as well as a variant DE. An effective method for identifying unknown photovoltaic model parameters was reported by Chen et al. [22] based on chaotic drifts in close proximity to the best agent and an opposition-based exploratory approach. Abbassi et al. [44] used an opposition-based learning modified salp swarm method (OLMSSA) to accurately identify the electrical equivalent circuit characteristics of the PV cell/two module’s diodes. For a more stable model and a more accurate estimation of the single diode PV model’s parameters, Ridha et al. [45] created a new HHO (BHHO) by combining random evolutionary stages from flower pollination algorithms with a powerful mutation strategy from DE with 2-Opt algorithms. For the detection of photovoltaic module parameters, Zhang et al. [46] presented an improved moth flame technique using an orthogonal learning approach. An improved HHO [47] may correctly forecast the characteristics of solar cells. One method for rapidly and precisely estimating photovoltaic model parameters has been proposed by Wang and colleagues [48], which utilizes the opposition-based learning mechanism in conjunction with the Nelder–Mead simplex methodology. For the solar cell system model, Fan et al. [49] suggested a unique DDSFLA method with a delayed dynamic step mechanism. By combining a gradient search method with an escape operator, Zhou and colleagues [50] came up with an improved gradient-based optimizer (RLGBO) for photovoltaic model parameter identification. In order to find unknown parameters in photovoltaic models, this novel technique (LCNMSE) combines Laplace’s cross-search mechanism with the Nelder–Mead simplex method [51]. In order to effectively and reliably estimate the unknown parameters of the photovoltaic model, according to Liu and colleagues, an enhanced slime mold algorithm that combines the Nelder–Mead simplex approach with the chaotic map has been proposed [52].

Teaching learning-based optimization (TLBO) was given by Patel et al. [53] for the extraction of solar cell parameters, and simulation results have proved its effectiveness on this topic. A hybrid of a TLBO and a DE for finding the parameters of a photovoltaic model has been tested and found to be effective [54]. Five parameters are extracted and the implicit voltage and current equations are solved concurrently, although only one diode Rsh-model is considered [55]. When it comes to finding the parameters of solar cells, Chen and his colleagues [56] have shown that the sine–cosine approach (SCA) with neighboring panels is an appropriate device. For this topic, Abdel-Basset et al. [57] developed an enhanced marine predators algorithm. Wang et al. [6] designed an ensemble multi-strategy framework by using shuffled frog-leading algorithms for this issue successfully.

However, we should keep in mind that despite the core ideal of the “no free lunch theory” (NFL) [58], metaheuristics cannot reject the benefits of deterministic parameter estimation techniques in terms of various factors that merit our attention. In the previous work, there is relatively little research on the PV parameters of single-crystal and double-crystal diodes at the same time. In addition, the research on the robustness of equipment exposed to a high voltage or harsh temperature is relatively insufficient. Alternatively, they may still be stuck in a state of search stagnation known as premature convergence; this problem is shown in current implementations of metaheuristics for PV cell parameter estimation by a relatively large value of root mean square error. Furthermore, the performance of some metaheuristic methods is dependent on predetermined parameters that must be appropriately set by users, leading to a high risk of stagnation and a weakening of their ability, which means that the majority of metaheuristic strategies can be added to extra special operators for various characteristics of complex and changeable problems. Furthermore, certain metaheuristic algorithm efficacies are reliant on user-defined parameters, which increase the possibility of stagnation and deterioration and some improvement room may still exist among some algorithms for this multimodal and nonlinear optimization problem. This study’s objective work on PV parameter recognition proof has the additional feature of being nonlinear and multimodal with several neighboring valleys, making it more difficult than other typical challenges. Stagnation is also more likely, posing a difficult dilemma for traditional approaches. Thus, it is necessary that developing an efficient and notable optimization technique for PV parameters estimation is still a problem worth studying.

The colony predation algorithm (CPA) was designed by Tu et al. [59]. CPA employs a mathematical mapping strategy similar to that of animal hunting parties, which includes distributing prey, surrounding prey, assisting the most probable successful hunter, and pursuing another target. To our knowledge, no study has used this approach to address photovoltaic model identification, although CPA has been applied to some practical cases [19]. In addition, the CPA’s property is not further investigated, and just a preliminary application of its original form is used for the issue. Therefore, this study will use CPA in this research to identify the optimal PV settings across multi-strategy learning mechanisms.

To further enhance the CPA’s capability for dealing with these issues, opposition-based learning and level-based learning are synchronously integrated into the original CPA, dubbed MLCPA, and they will be utilized to tackle PV parameter identification issues. To the best of our knowledge, opposition-based learning methodology and level-based learning are introduced into the original CPA for the first time. The original CPA’s capabilities may also be improved by using the greedy technique in conjunction with the specific behavior of opposition-based learning. Meanwhile, the level-based learning operator can assure a more balanced distribution of exploration and exploitation features, as well as more exploitative tendencies, while attempting to find a superior answer and a more potent means of exploitation. Additionally, the constructed MLCPA is being used for the first time to attempt to solve the parameter identification issue of transforming solar power into electrical power in this work.

To validate the MLCPA, a few benchmarks are used to determine the influence of these enhanced techniques, and then try to solve standard models, including SDM and DDM on a commercial R.T.C. France silicon sun-oriented cell with irradiation of 1000 W/m^2^ and a temperature of 33 °C. The 36 polycrystalline silicon cells are used in the Photowatt-PWP201 photovoltaic module, which has the same irradiance and a temperature of 45 °C. Additionally, to assess the proposed MLCPA robustness across a variety of temperature and light conditions, three distinct datasets derived from manufacturer information are used to see whether it is still capable of a good control transition in an open-air setting. The empirical evidence demonstrates that the suggested MLCPA approach may be capable of detecting the ideal photovoltaic parameters effectively. The contributions of this study are as follows:

- The well-known PV parameter identification situations are solved using an MLCPA ensemble. This study is the first to combine opposition-based learning and level-based learning in a single CPA-based strategy.
- In MLCPA, the greedy strategy is enhanced, and a better balance between exploratory and exploitative characteristics is assured for multi-strategy learning.
- The suggested MLCPA technique is used to estimate three PV parameters. The results of this study validate the MLCPA competitive and statistical advantages in determining unknown parameters for PV models.

The whole research is organized as follows: Section 2 contains extensive problem descriptions. The suggested method is shown in Section 3. Section 4 contains the experimental findings and analyses, which include benchmark results, photovoltaic cell and module results, and many manufacturer’s data sheets. Section 5 expands on the topic in Section 4 by discussing the outcomes. Finally, Section 6 discusses the conclusions and future work.

## 2. Definitions of Mathematical Model

### 2.1. Solar Cell

#### 2.1.1. SDM

The single diode model (SDM) is widely used to represent and realize the solar cell’s features. Using particular parts, the SDM model may be built. First, a current source is connected to a diode. Another resistor signifies the leakage current. Finally, a series resistor compensates for load current losses. The current of the SDM can be calculated by $I_{L}=I_{pht}-I_{d}-I_{sh}$ where $I_{L}$ stands for the ultimate output current, $I_{pht}$ is the photo-generated current, $I_{d}$ is the diode current estimated by $I_{d}=I_{sd}\cdot \left[\exp\left(\frac{q\cdot \left(V_{L}+R_{S}\cdot I_{L}\right)}{n\cdot k\cdot T}\right)-1\right]$, and $I_{sh}$ is the shunt resistor current calculated by $I_{sh}=\frac{V_{L}+R_{S}\cdot I_{L}}{R_{sh}}$. $R_{S}$ and $R_{sh}$ are shunt resistances, the two resistances in series. The output voltage is denoted by $V_{L}$, the reverse saturation current by $I_{sd}$, the diode ideality factor is *n*, the Boltzmann constant is *k* ($1.3806503\times 10^{-23}$ J/K), the magnitude of an electron’s charge is *q* ($1.60217646\times 10^{-19}$ C), and the cell temperature is T in Kelvin. Therefore, $I_{L}=I_{pht}-I_{d}-I_{sh}$ can be readjusted as follows:

(1) $$I_{L}=I_{pl_{r}}-I_{sdl}\cdot \left[\exp\left(\frac{q\cdot \left(V_{L}+R_{S}\cdot I_{L}\right)}{n\cdot k\cdot T}\right)-1\right]-\frac{V_{L}+R_{S}\cdot I_{L}}{R_{sh}}$$

where a single diode model has unknown parameters set $\left(I_{ph},I_{sd},R_{S},R_{sh},n\right)$, and an effective optimization method can identify these parameters accurately to reflect the performance of this solar cell.

#### 2.1.2. DDM

SDM is incapable of influencing the recombination of current misery within the exhaustion locality into consideration, but double diode models (DDM) are capable of doing so. The photo-generated current was shunted away from the photo-generated current source using two parallel diodes with a current source and shunt resistance in DDM. There are two devices in the circuit: one serves as a rectifier, while the other measures the charge recombination current and other circuit non-idealities. The output value is shown as follows:

(2) $$\begin{array}{c} \begin{array}{cc} \; & I_{L}=I_{ph}-I_{sd1}\cdot \left[\exp\left(\frac{q\cdot \left(V_{L}+R_{S}\cdot I_{L}\right)}{n_{1}\cdot k\cdot T}\right)-1\right]-\\ & I_{sd2}\cdot \left[\exp\left(\frac{q\cdot \left(V_{L}+R_{S}\cdot I_{L}\right)}{n_{2}\cdot k\cdot T}\right)-1\right]-\frac{V_{L}+R_{S}\cdot I_{L}}{R_{sh}} \end{array} \end{array}$$

where $I_{sd1}$ and $I_{Sd2}$ represent the saturation and diffusion currents. Both $n_{1}$ and $n_{2}$ include the ideality factors for diode dispersion and recombination. From this equation, it can be seen there are seven ambiguous parameters ($I_{ph},I_{sd1},I_{sd2},R_{S},R_{sh},n_{1},n_{2}$) in this DDM, which need to be identified for the solar cell’s fundamental operation.

### 2.2. PV Module Model

In practical terms, because of the layout and/or parallelism, the photovoltaic module displays fewer sunlight-based cells. The yield current can be calculated as follows:

(3) $$\begin{array}{c} \begin{array}{cc} \; & I_{L}/N_{p}=I_{ph}-I_{sd}\cdot \left[\exp\left(\frac{q\cdot \left(V_{L}/N_{S}+R_{S}\cdot I_{L}/N_{P}\right)}{n\cdot k\cdot T}\right)-1\right]\\ & -\frac{V_{L}/N_{S}+R_{S}\cdot I_{L}/N_{P}}{R_{sh}} \end{array} \end{array}$$

where $N_{p}$ and $N_{S}$ are the number of parallel sun-oriented cells, respectively. Similar to the single diode model, the mysterious parameters ($I_{sh},I_{sd},R_{S},R_{sh},n$) are necessary to be reached to improve the cases.

### 2.3. Objective Function Construction

In order to reduce the discrepancy between the photo-generated data and the estimated current data, we must find the optimal parameters for these solar cells. Photo-generated and calculated current data points have an error function given below:

(4) $$\begin{array}{c} \begin{array}{cc} \; & f_{k}\left(V_{L},I_{L},x\right)=I_{ph}-I_{sd}\cdot \left[\exp\left(\frac{q\cdot \left(V_{L}+R_{S}\cdot I_{L}\right)}{n\cdot k\cdot T}\right)-1\right]\\ & -\frac{V_{L}+R_{S}\cdot I_{L}}{R_{sh}}-I_{L}\\ \; & x=\left\{I_{ph},I_{sd},R_{S},R_{Sh},n\right\} \end{array} \end{array}$$

(5) $$\begin{array}{c} \begin{array}{cc} \; & f_{k}\left(V_{L},I_{L},x\right)=I_{ph}-I_{sd1}\cdot \left[\exp\left(\frac{q\cdot \left(V_{L}+R_{S}\cdot I_{L}\right)}{n_{1}\cdot k\cdot T}\right)-1\right]\\ & -I_{sd2}\cdot \left[\exp\left(\frac{q\cdot \left(V_{L}+R_{S}\cdot I_{L}\right)}{n_{2}\cdot k\cdot T}\right)-1\right]-\frac{V_{L}+R_{S}\cdot I_{L}}{R_{sh}}-I_{L}\\ \; & x=\left\{I_{ph},I_{sd1},I_{sd2},R_{S},R_{Sh},n_{1},n_{2}\right\} \end{array} \end{array}$$

To conduct a thorough evaluation of these photovoltaic modules, the root mean square error (RMSE) is used to quantify the contrast as in Equation (6), where *N* is the number of exploratory data points; this metric is sometimes referred to as the objection function [60]. As a result, in this case, the optimization strategy is focused on minimizing the root mean square error in conjunction with tweaking the arrangement vector *x* across all iterations.

(6) $$RMSE\left(x\right)=\sqrt{\frac{1}{N}\sum_{k=1}^{N}f_{k}{\left(V_{L},I_{L},x\right)}^{2}}\;k=1,2,\cdots ,N.$$

## 3. The Proposed CPA-Based Method

### 3.1. Summary of CPA

The colony predation algorithm was designed by Tu et al. [59] who employ a mathematical mapping strategy similar to that of animal hunting parties, which includes distributing prey, surrounding prey, assisting the most probable successful hunter, and pursuing another target. Individual cooperative communication and food-seeking behavior are represented by the following formulas:

(7) $${\vec{X}}_{j}^{i}\left(t+1\right)=r\cdot {\vec{X}}_{j}^{i_{1}}\left(t\right)+\left(1-r\right)\cdot \left(\left({\vec{X}}_{1}\left(t\right)+{\vec{X}}_{2}\left(t\right)\right)/2\right)$$

where *r* is a random value in [0, 1], ${\vec{X}}_{j}^{i_{1}}\left(t\right)$ is the target prey, and ${\vec{X}}_{1}$ and ${\vec{X}}_{2}$ are two individuals closed to the ${\vec{X}}_{j}^{i_{1}}\left(t\right)$, j∈1,2,…, dim. ${\vec{X}}_{j}^{i}\left(t+1\right)$ is the latest update position. The predation strategy is realized as $\vec{X}\left(t+1\right)={\vec{X}}_{\mathrm{best}\;}-S\cdot \left(r_{1}\left(ub-lb\right)+lb\right)$, where $\vec{X}\left(t+1\right)$ is the new individual at the ${\left(t+1\right)}^{th}$ iterations, ${\vec{X}}_{best\;}$ is the position of the food. *S* is the strength of prey and and its absolute value decreases with the number of assessments from *a* to 0, $r_{1}$ is the $\left[R_{1};R_{2};R_{3};\ldots R_{j}\right]$, j=dim represents the dimension of the population, ub, and lb represent the upper and lower bounds. *S* is calculated by $S=2\cdot S_{0}\cdot r_{2}-S_{0}$ and $S_{0}=a-t\cdot \left(\frac{a}{N}\right)$, where *N* is the number of individuals, $S_{0}$ reduces in value from a to 0, and t denotes the current number of evaluations and $r_{2}$ is a random value in [0, 1].

The hunting party will encircle the lone target and continue to approach it. This step may be mathematically described as follows:

(8) $$\vec{X}\left(t+1\right)={\vec{X}}_{best}-2\cdot S\cdot D\cdot e^{l}\cdot \tan\left(l\cdot \frac{\pi }{4}\right)$$

where *D* means the distance between the current individual and the prey *D*. The following mathematical formulas represent the odds of applying these two predatory strategies:

(9) $$\vec{X}\left(t+1\right)=\left\{\begin{array}{c} {\vec{X}}_{best}-S\cdot \left(r_{1}\cdot \left(ub-lb\right)+lb\right)r_{2}\geq 0.5\\ {\vec{X}}_{best}-2\cdot S\cdot D\cdot e^{l}\cdot \tan\left(l\cdot \frac{\pi }{4}\right)r_{2}<0.5 \end{array}\right.$$

Because the group may encounter difficulties when hunting monsters, the nearest individual seeks peer assistance. it can be expressed as $\vec{X}\left(t+1\right)={\vec{P}}_{\mathrm{nearest}\;}$, where the closest predator in the support group is indicated by ${\vec{P}}_{\mathrm{nearest}\;}$ nearest. $\vec{P}$ is the predator close to the prey. The search for the food can be:

(10) $$D_{1}=abs\left(2\cdot r_{4}\cdot {\vec{X}}_{\mathrm{rand}\;}-\vec{X}\left(t\right)\right)\vec{X}\left(t+1\right)={\vec{X}}_{\mathrm{rand}\;}-S\cdot D_{1}$$

where $D_{1}$ is the distance traveled by a random group, $r_{4}$ is generated randomly in [0, 1], and ${\vec{X}}_{\mathrm{rand}\;}$ is a random new individual. For more details, readers can refer to our original paper [59].

### 3.2. Multi-Strategy Learning

#### 3.2.1. Opposition-Based Learning

Opposition-based learning may help expedite the convergence of an algorithm, which is often used to find a global solution to a pending optimization issue that has not yet been solved. The opposite value of a number $X\in \left[L_{b},l_{u}\right]$ is calculated by $\bar{X}=ub+lb-X$, and lb and ub are the lowest and higher limits of the search space. For multidimensional decision space, $X_{i}=\left(X_{i1},X_{i2},X_{i3},\cdots ,X_{ij}\right)$ with $X_{ij}\in \left[l_{bj},l_{uj}\right],\;j=1,2,\cdots ,n$, the opposite point can be calculated as follows: ${\bar{X}}_{i}=\left({\bar{X}}_{i1},{\bar{X}}_{i2},{\bar{X}}_{i3},\cdots ,{\bar{X}}_{ij}\right),{\bar{X}}_{ij}=l_{bj}+l_{uj}-x_{ij}$. It was observed that when $f\left({\bar{X}}_{i}\right)$ is superior to $f\left(X_{i}\right)$, the opposing point $X_{i}$ may be replaced by the comparative arrangement ${\bar{X}}_{i}$. There are no changes that can be made if $f\left(X_{i}\right)$ continues to be superior to $f\left({\bar{X}}_{i}\right)$.

#### 3.2.2. Level-Based Learning

**Figure 1:**
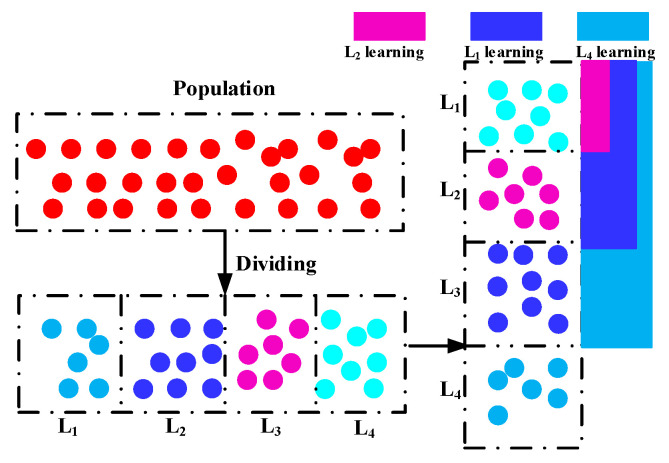
Framework of level-based learning strategy.

During the evolution process, individuals are often in a variety of distinct evolution states, each with a unique capability for exploring and exploiting the search space they are in. For the purpose of distinguishing between them, we first divide individuals into distinct levels based on their fitness ratings. To be specific, the number of *N* individuals can be divided into NL levels ($L_{1},L_{2},\cdots ,L_{NL}$) regarding their fitness. Then, in such levels, better individuals are assigned to higher levels, with a lower level index corresponding to a greater degree of quality. As a result, the highest level is $L_{1}$, and the lowest level is $L_{NL}$. In this study, the same number of individuals in each level is adopted, which is calculated by $L_{s}=N/NL$. In other words, individuals at a higher level often contain more advantageous information that may be used to drive the population toward the global optimal location. As a result, individuals at higher levels should direct those at lower levels to explore the whole solution space, allowing for rapid convergence and the discovery of interesting places. On the other hand, we observe individuals at various higher levels and discover that the higher the level to which an individual belongs, the more probable it is that the individual is near to the global optimal region. That is, individuals at various levels have varying degrees of exploitation strength. Similarly, individuals at various levels exhibit varying degrees of exploratory intensity. In other words, individuals with more exploitation potential often have less exploration potential, and vice versa. As a result, an individual at a lower level should learn from those at higher levels how to strike a balance between exploration and exploitation. The details of this idea can be seen in Figure 1. As seen in this picture, individuals at lower levels may possibly learn from particles at higher levels, and the number of possible exemplars for individuals at various levels varies. Specifically, when an individual’s level increases, it has fewer individuals at higher levels to learn from, which corresponds to the hypothesis that better individuals should engage in more exploitation than exploration. In general, this level-based learning may push individuals at lower levels to explore more and individuals at higher levels to exploit more.

### 3.3. Proposed MLCPA

In this study, the multi-strategy learning boosted colony predation algorithm (MLCPA) is designed by using opposition-based learning and level-based learning strategies synchronously. A more stable balance between the intensification and diversification characteristics of the original CPA can be achieved through the addition of opposition-based learning and level-based learning and then solving the parameter extraction of the photovoltaic problem. More specifically, the proposed MLCPA framework consists of three search stages, which are the colony predation algorithm stage, opposition-based learning, and level-based learning strategy. To our knowledge, the CPA is enhanced with these two learning strategies for the first time. The proposed MLCPA is composed of three parts. The first step is to set up the search population. The second is to finish the original CPA algorithm operators, and the third is to finish the multi-strategy learning operators. The detailed pseudocode of this proposed MLCPA can be seen in Algorithm 1. The original part of the CPA is executed first, and then the level-based learning is executed as shown in Algorithm 2, and the last part is opposition-based learning.
**Algorithm 1:** The pseudo code of MLCPA
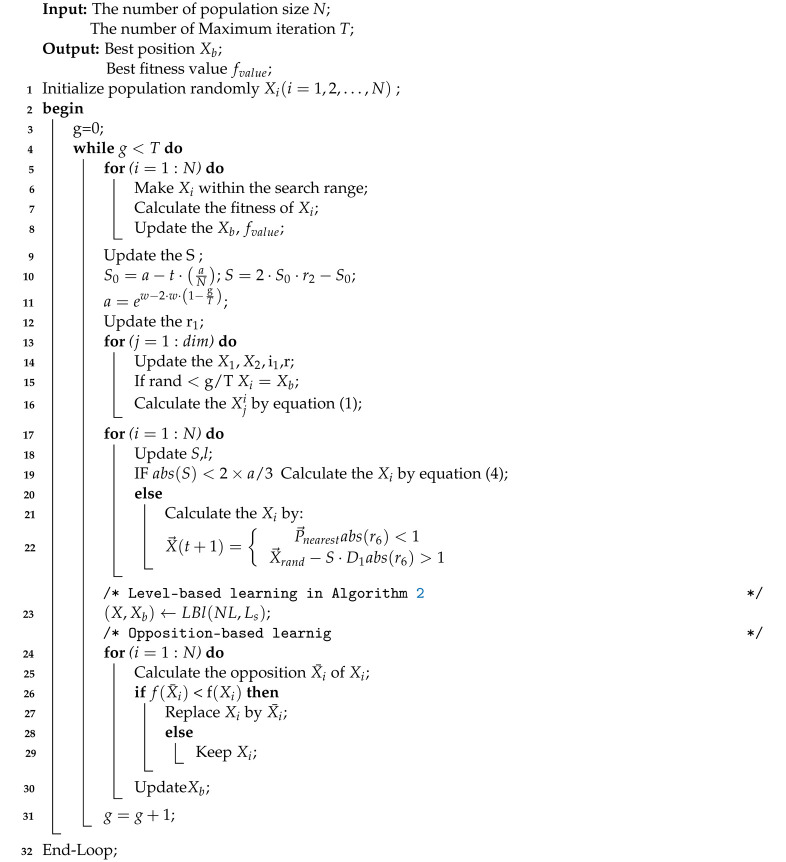

**Algorithm 2:** (LBl) Level-based learning
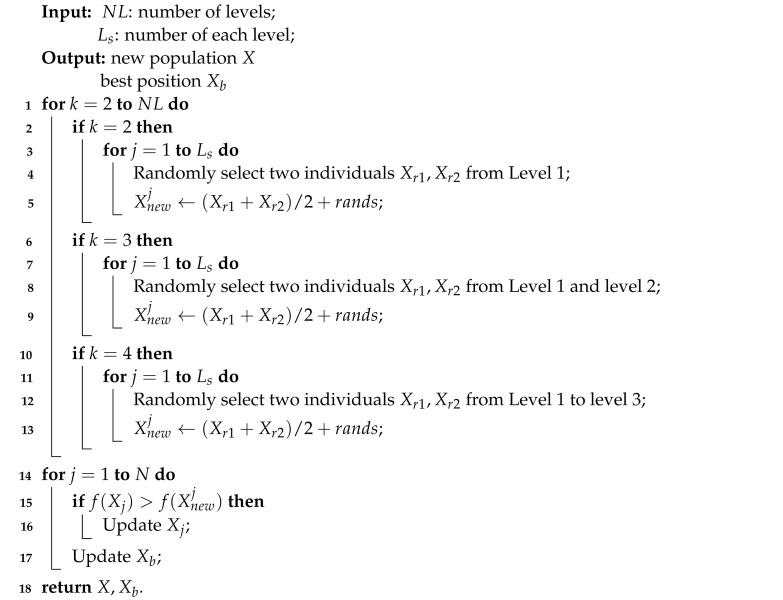



## 4. Numerical Results

An in-depth evaluation of the MLCPA method was carried out via three separate trials in this study. To begin, some benchmarks were performed to measure whether the MLCPA’s property with the multi-strategy learning operators was significantly better than that of the traditional CPA. There are seven unimodal tasks (F1–F7), six multimodal tasks (F8–F13), and ten different fixed-dimension multimodal tasks (F14–F23). Unlike unimodal tasks, which only have a single global ideal, multimodal and fixed-dimension multimodal tasks often have more than one local optimal value for assessing the exploratory attribute. In addition, MLCPA was compared to other current algorithms, including one variant of CPA, ECPA [19], the original CPA, BA [61], PSO [62], FA [63], and GSA [64] to further assess its performance, and the parameters of these algorithms are shown in Table 1.

For the second part, MLCPA is used to solve solar cells, including SDM, DDM, and cased PV modules. The same framework and computing conditions are used in this investigation, and the parameters’ limits are shown in Table 2 for comparison’s sake. Furtghermore, a few well-designed algorithms such as NPSOPC [65], BLPSO [66], CLPSO [67], IJAYA [68], GOTLBO [69], and EMSFLA [6] were all deployed as rivals. Additionally, the Wilcoxon signed-rank test with a significant degree of 0.05 is used in this study. The icons ‘+’, ‘−’, and ‘=’ indicate that MLCPA is essentially better than, more regretful than, or similar to its rival. To further assess the MLCPA’s stability in high-pressure or low-temperature conditions, three commonsense datasets from the manufacturer’s datasets were utilized in the third phase and this component also takes into account varied quantities of light and temperature.

The MATLAB R2020b platform is used to execute MLCPA and other competing algorithms in 30 separate runs to prevent the possibility of testing. A computer with Intel(R) Core(TM) i7-7500U CPU, 2.70 GHz, and 8.00 GB RAM is used in this study. For these photovoltaic examples, the maximum number of iterations is $1\times 10^{4}$, and the value of the root mean square error (RMSE) is considered as the fitness of each agent during the evolutionary process.

### 4.1. Results on Benchmarks

In this part, we compare the proposed MLCPA against other state-of-the-art approaches, including the original CPA, ECPA, BA, PSO, FA, and GSA. There are seven unimodal tasks (F1–F7), six multimodal tasks (F8–F13), and ten different fixed-dimension multimodal tasks (F14–F23). Table 3 summarizes the average outcomes (Avg), standard deviation (STD), and rank for dealing with F1–F23, with the average results of 30 independent executions and the bold one means that current algorithm has the best function value. It can be observed from this table that the proposed MLCPA can perform the best mean values among all these competitive algorithms on most benchmarks. Regarding the unimodal benchmarks, MLCPA demonstrates the best mean values as well as the standard deviation (STD) among these comparison algorithms, indicating that MLCPA can effectively solve the optimization problem with a single global optimum and that the multi-strategy learning operators can obviously improve the original CPA on unimodal tasks. Although MLCPA and ECPA may perform similarly on multimodal benchmarks, MLCPA continues to outperform all other algorithms on multimodal benchmarks. In terms of fixed-dimension multimodal tasks, the proposed MLCPA has a clear advantage over previous multimodal benchmark algorithms. In addition, the Friedman rank test is also recorded in Table 4; it can be seen that the proposed MLCPA has the best rank, and the ECPA has the second rank. Based on the above experimental statistics, a preliminary conclusion can be drawn that the performance of the algorithm proposed MLCPA in this paper has a significant improvement over the performance of the original CPA. The underlying reason behind this experimental phenomenon may be that the new fusion operator proposed has significantly improved the search performance of the algorithm, and can achieve a relatively good balance between intensification and diversification.

In order to further verify the property of the MLCPA, Table 3 displays the *p*-values of MLCPA and other techniques on F1–F23. This table demonstrates that the majority of *p*-values are less than 0.05, indicating that MLCPA has vastly superior properties to the original CPA, despite the fact that MLCPA may perform poorly under certain conditions. The MLCPA has the potential to estimate solar photovoltaic parameters due to its superior performance in a variety of complex optimization problems involving multiple peaks.

### 4.2. Results of Solar Cell

#### 4.2.1. Results on SDM

**Figure 2:**
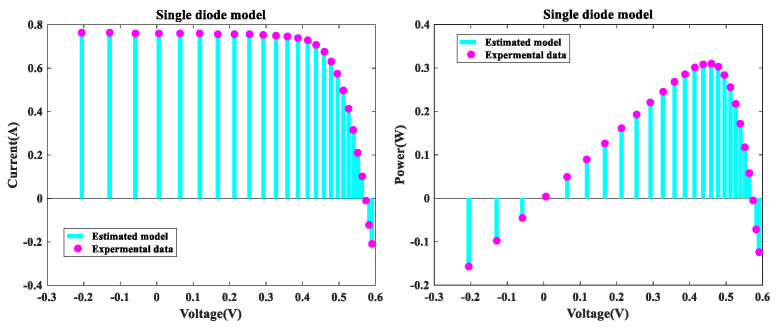
MLCPA for SDM I-V characteristics and P-V characteristics.

**Figure 3:**
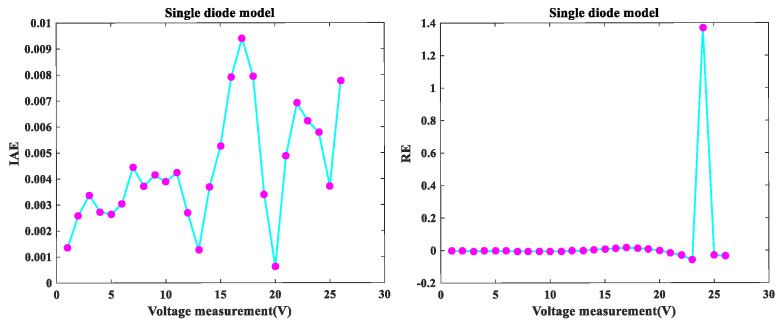
Error index values on SDM IAE values and RE values.

Figure 2 depicts the I-V and P-V fitting diagram of MLCPA for SDM. As noted, the I-V and P-V curves obtained from the model correspond well with experimental data over the whole voltage range. The error-index values for the computed electric current and the simulation test for this model are shown in IAE and RE values in Figure 3. It was determined to be less than 9.41557237E-03, and all RE values were between −5.668575E-02 and 1.368383E+00, indicating that the MLCPA is capable of substantially identifying the real attribute of SDM. Additionally, according to Table 5, there was an absolute individual error of 0.1058408 in this case, with a total of 0.0413232 inaccuracy between the trial and obtained data. These critical parameters in SDM can be clearly identified, and the provided technique MLCPA can also be completely supported on this topic.

Table 6 summarizes the results of 30 separate runs utilizing MLCPA and other competing algorithms. As can be seen, the MLCPA proposal has the lowest RMSE values among all comparator sets, indicating that its viability profile is more reliable and consistent than the other algorithms. Using statistical verification data, a significant performance gap between the proposed CPA-based algorithm and competing algorithms has been demonstrated. A 95% certainty level was used to estimate the confidence ranges for the parameter gauges, as shown in Table 7. It is apparent that MLCPA’s confidence interval has the best dispersion of any competitor method, indicating that it can successfully disentangle parameters in relatively accurate intermediate intervals.

#### 4.2.2. Results on DDM

**Figure 4:**
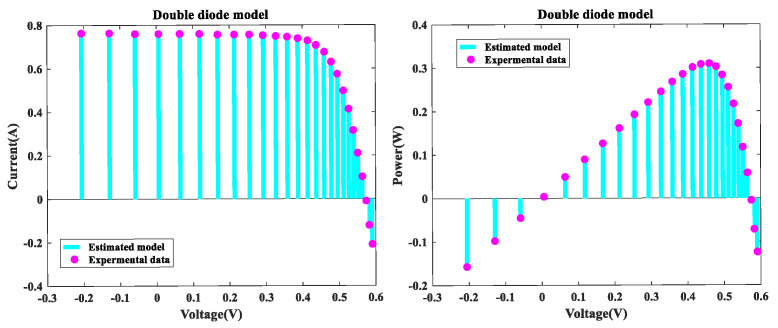
MLCPA for DDM I-V characteristics and P-V characteristics.

**Figure 5:**
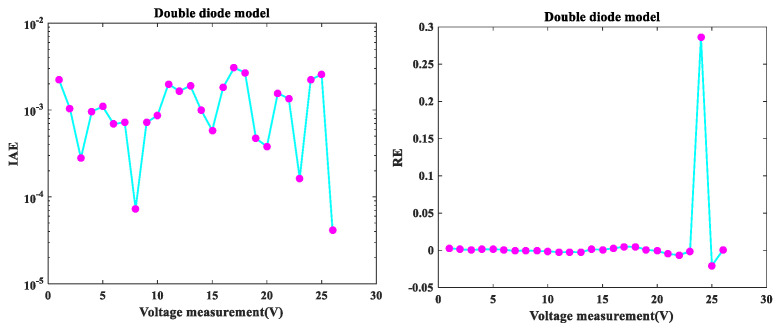
Error index values of DDM IAE values and RE values.

The MLCPA is evaluated in this part on the DDM issue. Figure 4 illustrates the I-V and P-V characteristics of MLCPA for DDM. The I-V and P-V curves produced by the proposed technique were both compatible with the trial data utilized in this inquiry throughout the whole voltage range. Figure 5 illustrates the error-index values for the regenerated electric current and the DDM test records with IAE and RE values. It was discovered that the maximum value of IAE was 3.058826E-03, while the top and lower limits of RE values were 2.8655865E-01 and −2.0446467E-02, respectively, indicating that the MLCPA can also accurately examine the DDM’s genuine property. The IAE’s factual records with the current and control values for DDM are disclosed in Table 8, and the sums of these values were 0.1058408 and 0.0413232, respectively. These critical parameters in DDM may be clearly identified, and the provided method’s MLCPA performance can also be properly supported in this instance.

As shown in Table 9, MLCPA continues to surpass its rivals in terms of root mean square error (RMSE) results for minimum (Min), mean (Avg), maximum (Max), and standard deviation (SD), despite the fact that other competitors may perform poorly in comparison. MLCPA outperformed other algorithms in the Wilcoxon signed-rank test. Additionally, the confidence interims for the parameter gauges were estimated to a degree of 95 percent, as shown in Table 10. MLCPA continued to be the most excellent interim certainty for each parameter with a degree of confidence of 95% and the least dispersion, as shown in this Table 10. Overall, the method suggested in this research is capable of properly interpreting the DDM model’s performance in order to make appropriate estimations.

#### 4.2.3. Results on PV Module

**Figure 6:**
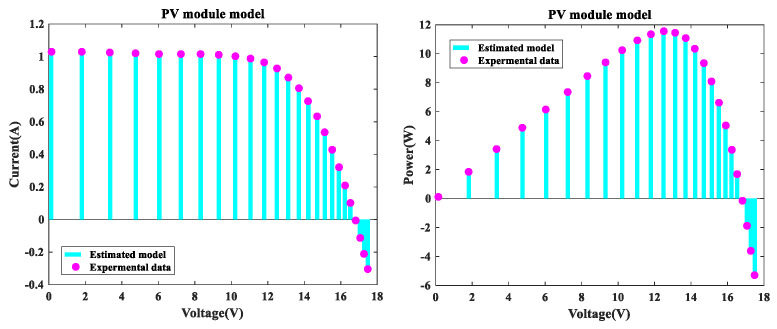
MLCPA for PV module single diode I-V characteristics and P-V characteristics.

**Figure 7:**
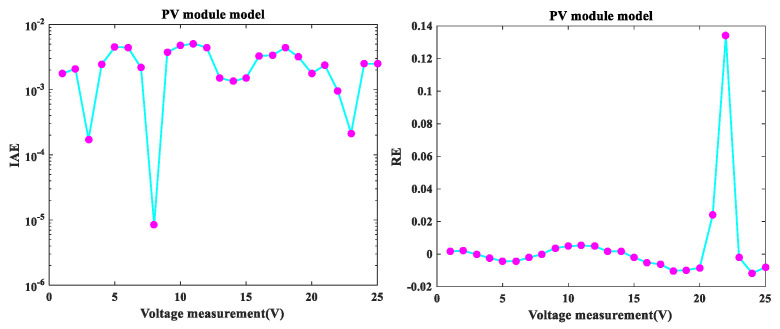
Error index of PV module (SDM) IAE values and RE values.

In this section, the MLCPA was used to address the issue of parameter estimation for the PV model example Photo Watt-PWP 201. As shown in Figure 6, across the entire voltage range, the MLCPA findings for I-V and P-V characteristics of the genuine data and the imitation records were in excellent agreement with the trial records. Additionally, Figure 7 shows the erroneous IAE and RE values. As seen in this figure, while the upper and lower limits for RE values were 1.34001E-01 (and −1.17285E-02, respectively), all IAE values were below 5.09229E-3. It can be concluded that MLCPA is capable of precisely measuring the real behavior of PV module modes. Additionally, Table 11 summarizes the IAE’s findings on PV mode, and the totals of these values were 0.06450231 and 0.752103455, respectively. Thus, it can be observed that the accuracy of the obtained parameters was adequate and of high quality, and the MLCPA’s performance on this issue was effectively confirmed.

Table 12 includes the exhaustive results of the MLCPA and several additional competitors for the photovoltaic module. As can be shown, the suggested MLCPA method surpassed its competition with a mean RMSE of 5.123650E-04 and a standard deviation of 1.4251600E-06 in this situation. BLPSO and NPSOPC may have had a comparable mean RMSE characteristic, however, EMSFLA ranked second in the findings and surpassed IJAYA and GOTLBO. In terms of the Wilcoxon signed-rank test, MLCPA greatly outperformed other algorithms in this PV situation, and in addition, the confidence interims for parameter gauges with a degree of certainty of 95 percent are also included in Table 13. Regarding this table, the MLCPA obtained the lowest dispersion for each parameter, demonstrating that this idea can properly extract the PV demonstration’s parameters, while SDM and DDM exhibit a similar pattern.

### 4.3. Results of the Manufacturer’s Datasheet

**Figure 8:**
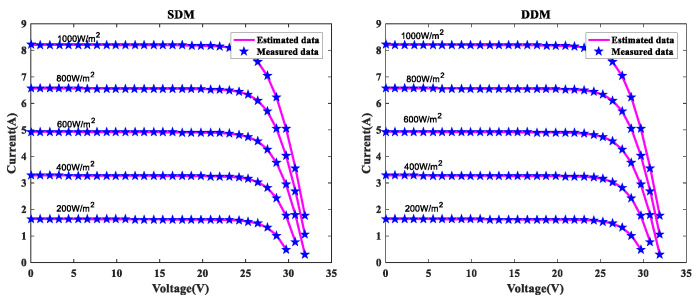
I-V for KC200GT at various irradiances.

**Figure 9:**
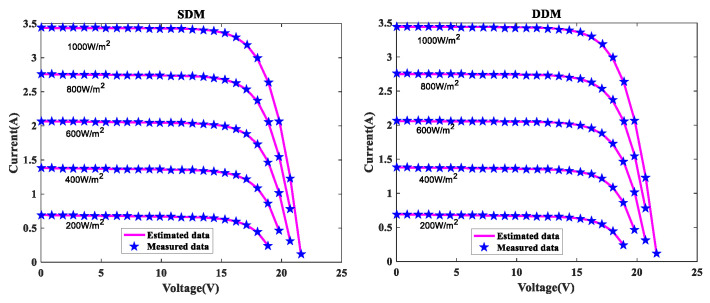
I-V characteristics for SM55 at various irradiances.

**Figure 10:**
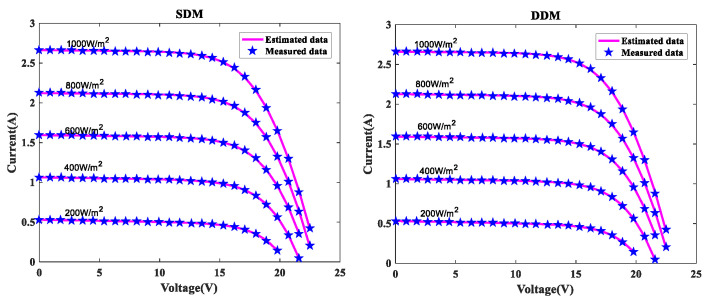
I-V characteristics for ST40 various irradiances.

**Figure 11:**
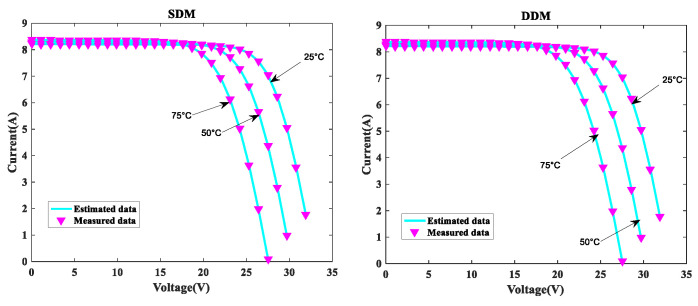
I-V for KC200GT at various temperatures.

**Figure 12:**
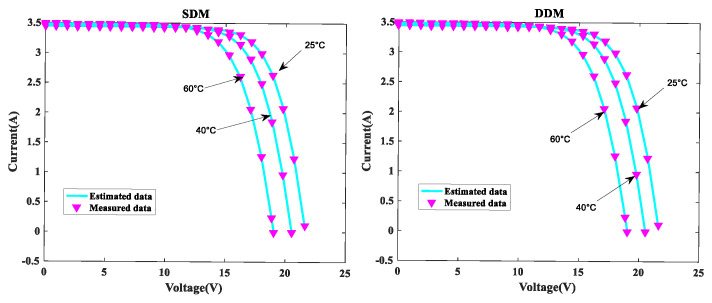
I-V for SM55 at various temperatures.

**Figure 13:**
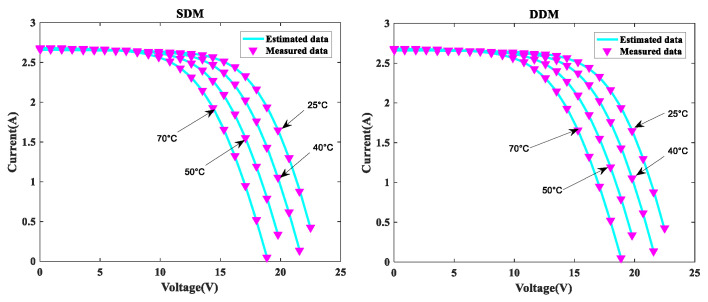
I-V for ST40 at various temperatures.

In order to further verify the suggested MLCPA approach, several manufacturer datasets were used in this part. These datasets included multicrystalline (KC200GT) [60] photovoltaic samples, monocrystalline (SM55) [70] and thin-film (ST40) [71] collected at various temperatures and irradiance levels. The stability and adaptability of the proposed algorithm MLCPA in a complex environment may be studied effectively by evaluating a solar devices’ capacity to recognize parameters at varying temperatures or pressures. In Table 14, Table 15 and Table 16, the optimal parameters for the single and double diode models of two solar modules at different levels of irradiance and constant temperature 25 °C were determined by rigorous testing and statistical analysis. In order to verify the accuracy of the obtained PV show parameters, the assessed electric current was obtained. The I-V characteristics of the two distinct modules are delineated at various levels of irradiance, specifically $200\;W/m^{2}$, $400\;W/m^{2}$, $600\;W/m^{2}$, $800\;W/m^{2}$, and $1000\;W/m^{2}$, as plotted in Figure 8, Figure 9 and Figure 10 respectively. To add to this, the ideal calculated parameters for these two PV modules at various temperatures and a constant level of $1000\;W/m^{2}$ irradiance are shown in Table 17, Table 18 and Table 19, as well as experimental and estimated (I-V) characteristics in Figure 11, Figure 12 and Figure 13.

Regarding the SDM, the I-V curves derived from these evaluated ideal parameters corresponded closely to the observed curves at varying temperatures and irradiance levels. MLCPA is able to guarantee an acceptable RMSE on the ST40, SM55, and KC200GT for SDM. Regarding the DDM module, it can be seen that the estimated results are also consistent with these datasets at various temperatures and irradiance levels; even in an unfavorable environment with severe irradiance or temperature levels, the MLCPA can still guarantee an acceptable RMSE value. On the basis of these results, it can be concluded that the proposed MLCPA can continue to provide a high-quality technique for parameter-recognizable proofs of solar cell modules even when subjected to a somewhat challenging environment, such as high pressure or extremely cold temperatures, and that the robustness of the proposed MLCPA has been successfully verified.

## 5. Discussions

Two learning-based operators were incorporated into the CPA in this work, based on CPA and using a multi-strategy learning approach. There was also extensive testing on the F1–F23 benchmarks, and the findings show that MLCPA’s capabilities have been significantly improved as a consequence of these new techniques being implemented. The estimated photovoltaic parameters using SDM, DDM, and PV, and three manufacturer’s datasheets were also subject to a thorough examination of MLCPA. Because of this, the suggested MLCPA algorithm should be taken into consideration for tackling the issue at hand as a viable alternative to the other competing algorithms.

According to Section 4.1, the suggested MLCPA was tested and compared against numerous existing competing algorithms on a variety of unimodal, multimodal, and fixed-dimension multimodal benchmarks. When compared to the original CPA, the MLCPA clearly outperformed it. Its properties were greatly enhanced and were superior to other algorithms. MLCPA was also used to identify parameters in these three interconnected modules. Observe that the MLCPA can precisely identify the crucial parameters of these issues, which are extremely discordant with recordings of experiments on these tasks throughout the complete voltage range. In terms of SDM, MLCPA has the best value among peers, with an RMSE of 3.254815E-04, a median RMSE of 3.254810E-04, and a mean RMSE of 5.421563E-04. According to the Wilcoxon test, MLCPA achieved the best DDM results (8.521426E-04 for the minimum RMSE, 8.854789E-04 for the medium RMSE, and 7.652418E-04 for the mean RMSE), beating out the competition’s findings by a wide margin. When it comes to PV modules, MLCPA showed the greatest RMSE values and was far better than other rivals in the Wilcoxon signed-rank test. In particular, the original CPA’s capacity may be improved in terms of the greedy technique of opposition-based learning. More exploitative tendencies in the search for the best solution, as well as a better exploitative ability when combined, can be guaranteed with level-based learning strategy additions. This means that, in comparison to other algorithms that can’t benefit from this characteristic, MLCPA may achieve an important capacity for exploiting. As a result, MLCPA’s primary physiognomies have advanced fundamentally, as shown by numerous factual statistics.

Additionally, three manufacturers’ datasets were utilized to study the MLCPA’s durability and effectiveness when faced with severe high-pressure or low-temperature conditions. In Section 4.3, detailed statistical results are shown clearly. When exposed to high temperatures such as 75 °C or high irradiation levels such as 1000 W/m^2^, the estimated current value may be determined using the ST40, SM55, and KC200GT data and a pleasing RMSE error was still available, showing that the MLCPA’s stability can be improved over time due to the introduced feature of multi-strategy learning. As a consequence, MLCPA may be regarded a potential approach for determining the characteristics of solar cells that display long-term stability in performance. The proposed optimizer MLCPA was not only studied for this research’s case studies; it may be used in other fields as well, such as power engineering. In the future, the MLCPA might be used to determine the appropriate settings for certain challenges. In addition, the research limitations of the algorithm proposed in this paper must also be noted. Firstly, this paper introduces new operators to the original CPA. As for the execution time of the algorithm, the computational time consumed was significantly higher than that of the original CPA, which is equivalent to sacrificing the computational time to replace the efficiency. Secondly, this paper only considers the single objective when designing algorithms, which is not enough for considering the application of robust algorithms. Finally, the dataset used in this study is very limited and open source. It is ideal to obtain data from real scenarios to verify the performance of the algorithm.

## 6. Conclusions and Future Works

In this paper, multi-strategy learning boosted colony predation algorithm (MLCPA) with opposition-based learning and level-based learning was used to extract the solar cell model parameters and photovoltaic modules. Based on the above experimental results, some conclusions can be drawn as follows:

- On benchmark results, the conventional CPA’s property can be enhanced through the unique behavior of opposition-based learning, and a more stable balance between intensification and diversification can be achieved through the addition of level-based learning.
- The results of the experiments show that MLCPA has a clear advantage over its rivals when it comes to estimating various PV parameters.
- Regarding results at different levels of temperature and irradiation, MLCPA can still perform very well even when it is under a lot of pressure or when the temperature is very low.
- The MLCPA–based approach may be employed as a viable device for resolving the knowledge of solar cell model parameters and photovoltaic modules, as well as providing an untapped option for solar energy conversion.

The proposed MLCPA has a few flaws that need to be investigated further in future developments. The propertiese of the MLCPA still have room to improve on, and new operators could be introduced into this MLCPA such as self-adaptive quasi-oppositional stochastic fractal search [72] or an elitism-based framework [73]. It is possible to create this proposal’s multi-objective variant. The multi-objective may, therefore, be solved by considering not only RMSE minimization but also another goal at the same time, and this multi-objective version may make advantage of additional application situations. Second, the binary version involving combinatorial optimization should be built while the real power dispatching management is still working on solving the electric power scheduling issues. As stated by the NFL, there is no omnipotent algorithm that can solve all optimization problems. It is thus necessary to create and propose an additional operator for MLCPA without stopping when confronted with a wide range of difficult and specialized situations.

## Figures and Tables

**Table 1:** Parameters setting.

Algorithm	Parameters					
MLCPA	T = 1 × 10^4^	N = 800	*r* ∈ [0, 1]	*r*_1_ ∈ [a, 0]	*l* ∈ [0, 1]	
ECPA	T = 1 × 10^4^	N = 800	*r* ∈ [0, 1]	*r*_1_ ∈ [a, 0]	*l* ∈ [0, 1]	
CPA	T = 1 × 10^4^	N = 800	*r* ∈ [0, 1]	*r*_1_ ∈ [a, 0]	*l* ∈ [0, 1]	
BA	T = 1 × 10^4^	N = 800	A = 1	*r*_0_ = 1	*α* = 0.97	*γ* = 0.1
FA	T = 1 × 10^4^	N = 800	*α* = 1	*β* = 1	*γ* = 1	*θ* = 0.97
PSO	T = 1 × 10^4^	N = 800	w = 0.73	*c*_1_ = 2	*c*_2_ = 1.45	
GSA	T = 1 × 10^4^	N = 800	*β* < 1	*r*_1_ ∈ [0, 1]	*ε* = 0.1	

**Table 2:** Parameters of PV cells.

Parameters	SDM/DDM		PV Module	
	Lower	Upper	Lower	Upper
$I_{ph}$ (A)	0	1	0	2
$I_{sd},I_{sd1},I_{sd2}$ (μA)	0	1	0	50
$R_{s}\;\left(\Omega \right)$	0	0.5	0	2
$R_{sh}\;\left(\Omega \right)$	0	100	0	2000
$n,n_{1},n_{2}$	1	2	1	50

**Table 3:** Results of benchmark functions.

Algorithm	F1		F2		F3		F4		F5	
	Mean	STD	Mean	STD	Mean	STD	Mean	STD	Mean	STD
**MLCPA**	**5.75527E-27**	**1.36464E-26**	**1.04376E-13**	**3.00934E-13**	**5.840936179**	**9.51954766**	**2.58099198**	**0.203471343**	**64.37797939**	**38.83994805**
**ECPA**	0.065851962	0.01450545	0.04233395	0.007668204	25,428.93541	4439.738486	33.88530958	1.791606316	1033.347968	310.9714381
**CPA**	3.08753E-08	6.52446E-09	4.254124125	3.514089388	1822.144757	726.2976603	17.52082219	5.458481451	304.9387696	423.3078379
**BA**	0.799583291	0.658936455	400.7877089	940.0303528	100.7379693	231.9778623	25.5102961	8.101399159	461.7819339	361.9312339
**PSO**	341.7327582	18.77461198	4,367,308.91	1,2801,797.81	2009.730348	231.2372669	6.564342552	0.498086347	583,237.9647	82,668.39033
**FA**	27,674.06498	1917.24971	1675.747384	4578.436322	67,493.80174	6067.491746	55.70068878	3.055311113	29,784,606.34	6,892,284.749
**GSA**	27,674.06498	2.122814682	24.02760791	1.604394227	1908.273815	354.8591782	19.92782253	4.079685329	25,324.73663	8645.662231
**Algorithm**	**F6**		**F7**		**F8**		**F9**		**F10**	
	**Mean**	**STD**	**Mean**	**STD**	**Mean**	**STD**	**Mean**	**STD**	**Mean**	**STD**
**MLCPA**	**1.14442E-26**	**3.61893E-26**	**0.052774466**	**0.01101405**	−20,072.70069	414.8160713	**1.890421201**	**2.172262392**	2.635387646	1.261668175
**ECPA**	0.077348916	0.027514294	0.075555579	0.038190439	**−20,841.77987**	**117.7530467**	6.412525374	2.230915945	**0.519097857**	**0.224388778**
**CPA**	3.25987E-08	8.7377E-09	0.18020436	0.055115469	−12,136.69957	977.9850953	130.2398018	33.7517942	3.498668724	1.014439626
**BA**	0.680151694	0.44371473	75.88375881	33.40529498	−12,266.0308	728.1323577	515.231117	42.93164387	2.759925245	0.459584906
**PSO**	345.5150367	32.75316191	458.8228114	60.79687443	−11,184.28357	2843.601202	701.3167648	35.94952768	9.676076642	0.223571279
**FA**	27,154.49353	1137.327961	24.29908509	3.657162415	−5013.289513	442.6617052	477.7876516	17.53403846	17.11461998	0.339628567
**GSA**	29.40259484	2.894543722	144.1775481	14.40933244	−3288.89598	513.4780641	388.6582843	13.16162903	4.405353994	0.137453874
**Algorithm**	**F11**		**F12**		**F13**		**F14**		**F15**	
	**Mean**	**STD**	**Mean**	**STD**	**Mean**	**STD**	**Mean**	**STD**	**Mean**	**STD**
**MLCPA**	**0.001231762**	**0.003894698**	0.904426211	1.345016196	**0.239508564**	**0.070622611**	**0.998003838**	**0**	**0.000307486**	**1.87789E-19**
**ECPA**	0.106220178	0.024503231	**0.004343884**	**0.002364662**	1.055038347	2.519187764	0.998003838	0	0.000624949	4.98844E-05
**CPA**	0.009107612	0.009353361	8.755187402	1.682931012	34.94612703	16.51345456	0.998003838	1.04673E-16	0.000813065	0.000236164
**BA**	68.38368022	44.82050201	14.20880511	4.926597063	0.336381494	0.152980642	3.17032675	2.265805091	0.006789254	0.009371627
**PSO**	1.084715087	0.007965212	8.191463023	1.147336558	714.6723147	756.3122626	3.359077361	3.134197666	0.001098543	0.00013014
**FA**	242.5413774	17.35652539	20,753,851.41	6,542,063.198	80,548,261.46	17,529,188.22	0.998366158	0.000326668	0.001519523	0.000382074
**GSA**	0.519157425	0.050475316	0.609869705	0.122426054	27.37365146	7.078467691	1.006728077	0.0125747	0.001800668	0.000482029
**Algorithm**	**F16**		**F17**		**F18**		**F19**		**F20**	
	**Mean**	**STD**	**Mean**	**STD**	**Mean**	**STD**	**Mean**	**STD**	**Mean**	**STD**
**MLCPA**	**−1.031628453**	**0**	**0.397887358**	**0**	**3**	0	**−3.862782148**	**9.36222E-16**	−3.286327235	0.057430834
**ECPA**	−1.031628453	1.4803E-16	0.397887358	0	3	2.96059E-16	−3.862782148	9.36222E-16	**−3.321995172**	**1.27622E-10**
**CPA**	−1.031628453	2.15535E-15	0.397887358	1.4014E-15	3	2.13599E-14	−3.862782148	3.89124E-15	−3.214855727	0.037645246
**BA**	−1.031277488	0.000274288	0.39840234	0.000347621	3.044705684	0.046499919	−3.846544147	0.007972701	−2.883082762	0.116566175
**PSO**	−1.031002898	0.000829827	0.39855564	0.000809338	3.081066859	0.072669656	−3.855097484	0.00574293	−2.891382261	0.179745644
**FA**	−1.031170217	0.00018459	0.398413906	0.00047803	3.010790018	0.009277712	−3.861733822	0.000603663	−3.225119683	0.051217841
**GSA**	−1.030229601	0.001223355	0.398995504	0.001237215	3.025016902	0.027518762	−3.860375701	0.001878432	−3.091128646	0.045999534
**Algorithm**	**F21**		**F22**		**F23**		**Ranks**			
	**Mean**	**STD**	**Mean**	**STD**	**Mean**	**STD**				
**MLCPA**	**−10.12039243**	**0.103151497**	−7.443963573	3.830028648	**−10.53639948**	**2.00069E-05**	**1**			
**ECPA**	−8.395680243	2.905383344	**−10.40277518**	**0.000268639**	−9.054843936	3.14113854	**2**			
**CPA**	−8.637471417	2.440554365	−10.40294057	6.13478E-12	−9.769883106	2.423970295	**5**			
**BA**	−6.911352163	2.310719511	−6.600588026	2.205927435	−7.555515685	2.763619922	**3**			
**PSO**	−3.807928927	0.734765998	−5.606258396	1.227741003	−5.475705542	1.818534293	**4**			
**FA**	−6.646041381	1.210477489	−7.471325706	0.695523191	−7.718999567	1.157529954	**6**			
**GSA**	−4.410286194	0.975998272	−4.641430824	1.673804469	−4.513629727	1.098803484	**7**			

**Table 4:** Results of *p*-values for MLCPA and other algorithms.

F	Algorithm					
	ECPA	CPA	BA	PSO	FA	GSA
**F1**	0.001953125+	0.001953125+	0.001953125+	0.001953125+	0.001953125+	0.001953125+
**F2**	0.001953125+	0.001953125+	0.001953125+	0.001953125+	0.001953125+	0.001953125+
**F3**	0.001953125+	0.001953125+	0.009765625+	0.001953125+	0.001953125+	0.001953125+
**F4**	0.001953125+	0.000000375+	0.083984375+	0.001953125+	0.001953125+	0.001953125+
**F5**	0.001953125+	0.064453125+	0.001953125+	0.001953125+	0.001953125+	0.001953125+
**F6**	0.001953125+	0.001953125+	0.001953125+	0.001953125+	0.001953125+	0.001953125+
**F7**	0.001953125+	0.00390625+	0.001953125+	0.001953125+	0.001953125+	0.001953125+
**F8**	0.001953125+	0.001953125+	0.001953125+	0.001953125+	0.001953125+	0.001953125+
**F9**	0.009765625+	0.001953125+	0.001953125+	0.001953125+	0.001953125+	0.001953125+
**F10**	0.0601953125−	0.001005468+	0.006953125+	0.001953125+	0.001953125+	0.005859375+
**F11**	0.001953125+	0.083984375+	0.001953125+	0.001953125+	0.001953125+	0.001953125+
**F12**	0.0803984375−	0.001953125+	0.001953125+	0.001953125+	0.001953125+	0.007695312+
**F13**	0.001953125+	0.001953125+	0.008457031+	0.001953125+	0.001953125+	0.001953125+
**F14**	0.001953125+	0.000000005+	0.001953125+	0.001953125+	0.001953125+	0.001953125+
**F15**	0.001953125+	0.001953125+	0.001953125+	0.001953125+	0.001953125+	0.001953125+
**F16**	0.001953125+	0.00390625+	0.001953125+	0.001953125+	0.001953125+	0.001953125+
**F17**	0.001953125+	0.00003125+	0.001953125+	0.001953125+	0.001953125+	0.001953125+
**F18**	0.001953125+	0.001953125+	0.001953125+	0.001953125+	0.001953125+	0.001953125+
**F19**	0.001953125+	0.001953125+	0.001953125+	0.001953125+	0.001953125+	0.001953125+
**F20**	0.54000006875−	0.001953125+	0.001953125+	0.001953125+	0.064453125+	0.001953125+
**F21**	0.0091015625+	0.000322265+	0.000003750+	0.00390625+	0.048828125+	0.005859375+
**F22**	0.54001015625−	0.000556640+	0.000006250+	0.006445312+	0.006953125+	0.003515625+
**F23**	0.00431640625+	0.000000375+	0.002324218+	0.037109375+	0.003222656+	0.005859375+

**Table 5:** IAE of MLCPA on SDM.

Item	Measured Data		Simulated Current Data		Simulated Power Data	
	V(V)	I(A)	$I_{\mathrm{sim}}\left(A\right)$	${\mathrm{IAE}}_{I}\left(A\right)$	$P_{\mathrm{sim}}\left(W\right)$	${\mathrm{IAE}}_{P}\left(A\right)$
**1**	−0.2057	0.764	0.7653501	0.0013501	−0.157433	0.0002777
**2**	−0.1291	0.762	0.7645838	0.0025838	−0.098708	0.0003336
**3**	−0.0588	0.7605	0.7638798	0.0033798	−0.044916	0.0001987
**4**	0.0057	0.7605	0.7632307	0.0027307	0.0043504	1.556E-05
**5**	0.0646	0.76	0.762629	0.002629	0.0492658	0.0001698
**6**	0.1185	0.759	0.7620513	0.0030513	0.0903031	0.0003616
**7**	0.1678	0.757	0.7614508	0.0044508	0.1277714	0.0007468
**8**	0.2132	0.757	0.7607189	0.0037189	0.1621853	0.0007929
**9**	0.2545	0.7555	0.7596598	0.0041598	0.1933334	0.0010587
**10**	0.2924	0.754	0.7578794	0.0038794	0.2216039	0.0011343
**11**	0.3269	0.7505	0.7547409	0.0042409	0.2467248	0.0013863
**12**	0.3585	0.7465	0.7491864	0.0026864	0.2685833	0.0009631
**13**	0.3873	0.7385	0.739754	0.001254	0.2865067	0.0004857
**14**	0.4137	0.728	0.724325	0.003675	0.2996532	0.0015204
**15**	0.4373	0.7065	0.7012516	0.0052484	0.3066573	0.0022951
**16**	0.459	0.6755	0.6675943	0.0079057	0.3064258	0.0036287
**17**	0.4784	0.632	0.6225844	0.0094156	0.2978444	0.0045044
**18**	0.496	0.573	0.5650594	0.0079406	0.2802695	0.0039385
**19**	0.5119	0.499	0.4955979	0.0034021	0.2536966	0.0017415
**20**	0.5265	0.413	0.4136181	0.0006181	0.2177699	0.0003254
**21**	0.5398	0.3165	0.3213822	0.0048822	0.1734821	0.0026354
**22**	0.5521	0.212	0.2189112	0.0069112	0.1208609	0.0038157
**23**	0.5633	0.1035	0.1097195	0.0062195	0.061805	0.0035035
**24**	0.5736	−0.01	−0.004222	0.0057777	−0.002422	0.0033141
**25**	0.5833	−0.123	−0.12673	0.0037299	−0.073922	0.0021756
**26**	0.59	−0.21	−0.217761	0.0077611	−0.128479	0.004579
**Sun of IAE**	NA	NA	NA	0.1058408	NA	0.0413232

**Table 6:** RMSE of SDM.

Algorithm	RMSE					
	Min	Median	Mean	Max	SD	Sig
NPSOPC	7.563212E-04	8.524334E-04	9.585124E-04	9.874523E-04	2.356454E-04	**+**
BLPSO	1.102051E-03	1.570244E-03	1.507212E-03	2.205341E-03	2.544534E-04	**+**
CLPSO	1.031352E-03	1.073221E-03	1.121336E-03	1.410112E-03	5.566321E-05	**+**
IJAYA	1.332253E-03	1.494334E-03	1.312122E-03	1.761221E-03	2.541251E-04	**+**
GOTLBO	1.333214E-03	1.213214E-03	1.041044E-03	2.363254E-03	1.320325E-03	**+**
EMSFLA	5.423012E-04	4.312034E-04	6.213425E-04	6.513213E-04	1.110321E-06	**+**
MLCPA	3.254815E-04	3.254810E-04	5.421563E-04	6.895214E-04	1.012365E-06	

**Table 7:** Confidence intervals on SDM.

Parameters	Algorithm						
	NPSOPC	BLPSO	CLPSO	IJAYA	GOTLBO	EMSFLA	MLCPA
$I_{ph}\;\left(A\right)$	(0.522 0.695)	(0.532 0.683)	(0.633 0.723)	(0.682 0.742)	(0.655 0.755)	(0.763 0.773)	(0.764 0.770)
$I_{sd}\;\left(\mu A\right)$	(0.202 0.463)	(0.213 0.457)	(0.209 0.431)	(0.231 0.322)	(0.292 0.452)	(0.321 0.325)	(0.322 0.324)
$R_{S}\;\left(\Omega \right)$	(0.014 0.095)	(0.014 0.055)	(0.019 0.060)	(0.023 0.051)	(0.033 0.058)	(0.026 0.039)	(0.026 0.032)
$R_{sh}\;\left(\Omega \right)$	(46.52 58.52)	(48.49 57.52)	(48.28 54.54)	(48.22 57.32)	(40.52 61.35)	(53.71 54.89)	(53.74 54.79)
*n*	(1.022 1.679)	(1.032 1.583)	(1.224 1.684)	(1.011 1.492)	(1.224 1.351)	(1.112 1.351)	(1.113 1.350)

**Table 8:** IAE of MLCPA on DDM.

Item	Measured Data		Simulated Current Data		Simulated Power Data	
	V(V)	I(A)	$I_{\mathrm{sim}}\left(A\right)$	${\mathrm{IAE}}_{I}\left(A\right)$	$P_{\mathrm{sim}}\left(W\right)$	${\mathrm{IAE}}_{P}\left(A\right)$
**1**	−0.2057	0.764	0.7617736	0.002226441	−0.156697	0.000457979
**2**	−0.1291	0.762	0.7609644	0.001035643	−0.09824	0.000133701
**3**	−0.0588	0.7605	0.7602211	0.000278939	−0.044701	1.64016E-05
**4**	0.0057	0.7605	0.7595368	0.00096323	0.0043294	5.49041E-06
**5**	0.0646	0.76	0.7589064	0.00109363	0.0490254	7.06485E-05
**6**	0.1185	0.759	0.7583131	0.000686857	0.0898601	8.13925E-05
**7**	0.1678	0.757	0.7577266	0.000726557	0.1271465	0.000121916
**8**	0.2132	0.757	0.7570731	7.30649E-05	0.161408	1.55774E-05
**9**	0.2545	0.7555	0.7562184	0.000718387	0.1924576	0.000182829
**10**	0.2924	0.754	0.7548652	0.000865189	0.2207226	0.000252981
**11**	0.3269	0.7505	0.7524914	0.001991435	0.2459895	0.000651
**12**	0.3585	0.7465	0.7481506	0.001650619	0.268212	0.000591747
**13**	0.3873	0.7385	0.7404063	0.001906322	0.2867594	0.000738318
**14**	0.4137	0.728	0.7270074	0.000992558	0.300763	0.000410621
**15**	0.4373	0.7065	0.7059163	0.000583722	0.3086972	0.000255262
**16**	0.459	0.6755	0.6736805	0.00181951	0.3092193	0.000835155
**17**	0.4784	0.632	0.6289412	0.003058826	0.3008855	0.001463342
**18**	0.496	0.573	0.5702996	0.002700437	0.2828686	0.001339417
**19**	0.5119	0.499	0.4985288	0.000471243	0.2551969	0.000241229
**20**	0.5265	0.413	0.4133805	0.000380477	0.2176448	0.000200321
**21**	0.5398	0.3165	0.3180657	0.001565708	0.1716919	0.000845169
**22**	0.5521	0.212	0.2133478	0.001347796	0.1177893	0.000744118
**23**	0.5633	0.1035	0.1036641	0.000164109	0.058394	9.24425E-05
**24**	0.5736	−0.01	−0.007773	0.002227327	−0.004458	0.001277595
**25**	0.5833	−0.123	−0.125567	0.00256741	−0.073243	0.00149757
**26**	0.59	−0.21	−0.209959	4.11952E-05	−0.123876	2.43052E-05
**Sun of IAE**	NA	NA	NA	0.029910188	NA	0.01254653

**Table 9:** RMSE of DDM.

Algorithm	RMSE					
	Min	Median	Mean	Max	SD	Sig
NPSOPC	7.658E-0.4	8.521432E-04	9.320841E-04	9.689725E-04	3.254325E-04	**+**
BLPSO	1.014332E-03	1.103213E-03	1.151814E-03	1.521562E-03	1.657213E-04	**+**
CLPSO	1.021230E-03	1.093654E-03	1.121236E-03	1.402823E-03	5.442184E-04	**+**
IJAYA	1.352120E-03	1.594544E-03	1.792723E-03	1.964354E-03	1.434545E-04	**+**
GOTLBO	1.303320E-03	1.131253E-03	1.045332E-03	4.433423E-03	1.043424E-04	**+**
EMSFLA	9.723545E-04	9.781236E-04	9.328255E-04	9.689824E-04	1.432190E-06	**+**
MLCPA	8.521426E-04	8.854789E-04	7.652418E-04	9.452365E-04	1.251485E-06	

**Table 10:** Confidence intervals on DDM.

Parameters	Algorithm						
	NPSOPC	BLPSO	CLPSO	IJAYA	GOTLBO	EMSFLA	MLCPA
$I_{ph}\;\left(A\right)$	(0.632 0.785)	(0.676 0.792)	(0.480 0.765)	(0.595 0.798)	(0.699 0.775)	(0.748 0.758)	(0.750 0.757)
$I_{sd1}\;\left(\mu A\right)$	(0.650 0.852)	(0.582 0.746)	(0.678 0.776)	(0.632 0.833)	(0.722 0.795)	(0.746 0.760)	(0.748 0.758)
$I_{sd2}\;\left(\mu A\right)$	(0.123 0.341)	(0.137 0.321)	(0.142 0.242)	(0.179 0.268)	(0.179 0.244)	(0.215 0.235)	(0.216 0.237)
$R_{S}\;\left(\Omega \right)$	(0.010 0.58)	(0.030 0.45)	(0.023 0.53)	(0.033 0.052)	(0.033 0.053)	(0.034 0.040)	(0.035 0.040)
$R_{sh}\;\left(\Omega \right)$	(38.78 56.42)	(38.78 55.44)	(48.46 57.54)	(49.66 59.88)	(49.54 58.54)	(55.42 55.59)	(55.43 55.58)
$n_{1}$	(1.112 1.854)	(1.213 1.653)	(1.004 1.587)	(1.015 1.568)	(1.003 1.585)	(1.035 1.451)	(1.037 1.450)
$n_{2}$	(1.018 1.638)	(1.028 1.553)	(1.328 1.922)	(1.226 1.743)	(1.031 1.625)	(1.202 1.388)	(1.212 1.382)

**Table 11:** IAE of MLCPA on PV module.

Item	Measured Data		Simulated Current Data		Simulated Power Data	
	V(V)	I(A)	$I_{\mathrm{sim}}\left(A\right)$	${\mathrm{IAE}}_{I}\left(A\right)$	$P_{\mathrm{sim}}\left(W\right)$	${\mathrm{IAE}}_{P}\left(A\right)$
**1**	0.1248	1.0315	1.0297422	0.001757828	0.128511823	0.000219377
**2**	1.8093	1.03	1.027908	0.002091969	1.859794	0.003785
**3**	3.3511	1.026	1.0261732	0.000173151	3.438808848	0.000580248
**4**	4.7622	1.022	1.0244346	0.002434581	4.878562361	0.011593961
**5**	6.0538	1.018	1.022493	0.004493033	6.189968321	0.027199921
**6**	7.2364	1.0155	1.0199641	0.004464082	7.380868085	0.032303885
**7**	8.3189	1.014	1.0161659	0.002165872	8.453382275	0.018017675
**8**	9.3097	1.01	1.0099915	8.45106E-06	9.402718323	7.86769E-05
**9**	10.2163	1.0035	0.9997481	0.003751934	10.21372616	0.038330888
**10**	11.0449	0.988	0.9832677	0.004732287	10.86009356	0.052267638
**11**	11.8018	0.963	0.9579077	0.005092289	11.30503522	0.060098178
**12**	12.4929	0.9255	0.9210776	0.004422369	11.50693074	0.055248211
**13**	13.1231	0.8725	0.8709793	0.001520728	11.42994808	0.019956671
**14**	13.6983	0.8075	0.8061617	0.001338282	11.04304506	0.018332191
**15**	14.2221	0.7265	0.7280164	0.001516423	10.35392237	0.021566719
**16**	14.6995	0.6345	0.6377934	0.003293432	9.375244548	0.048411798
**17**	15.1346	0.5345	0.5378621	0.00336212	8.140328046	0.050884346
**18**	15.5311	0.4275	0.431932	0.004432	6.708379092	0.068833842
**19**	15.8929	0.3185	0.3216599	0.003159944	5.11210933	0.05022068
**20**	16.2229	0.2085	0.2102778	0.001777847	3.411316482	0.028841832
**21**	16.5241	0.101	0.0986204	0.002379619	1.629613037	0.039321063
**22**	16.7987	−0.008	−0.0070547	0.00094533	−0.118509279	0.015880321
**23**	17.0499	−0.111	−0.1112143	0.000214256	−1.896191944	0.003653044
**24**	17.2793	−0.209	−0.2114803	0.002480349	−3.654232399	0.042858699
**25**	17.4885	−0.303	−0.3054941	0.00249413	−5.342634089	0.043618589
**Sun of IAE**	NA	NA	NA	0.06450231	NA	0.752103455

**Table 12:** RMSE values of PV.

Algorithm	RMSE					
	Min	Median	Mean	Max	SD	Sig
**NPSOPC**	1.235823E-03	1.563242E-03	2.415636E-03	2.684511E-03	2.421432E-04	**+**
**BLPSO**	1.203422E-03	1.634324E-03	1.482435E-03	2.205422E-03	2.337654E-04	**+**
**CLPSO**	1.205313E-03	1.214023E-03	1.131923E-03	2.313454E-03	3.457322E-04	**+**
**IJAYA**	1.223914E-03	1.533423E-03	1.212984E-03	2.718421E-03	2.545321E-04	**+**
**GOTLBO**	1.501315E-03	1.313242E-03	1.213414E-03	2.214313E-03	2.454324E-04	**+**
**EMSFLA**	6.301510E-04	5.353200E-04	6.534320E-04	7.878410E-04	1.631410E-06	**+**
**MLCPA**	4.210151E-04	4.352112E-04	5.123650E-04	6.521489E-04	1.425160E-06	

**Table 13:** Confidence intervals on PV.

Parameters	Algorithm						
	NPSOPC	BLPSO	CLPSO	IJAYA	GOTLBO	EMSFLA	MLCPA
$I_{ph}\;\left(A\right)$	(0.583 0.764)	(0.669 0.792)	(0.586 0.763)	(0.591 0.715)	(0.692 0.781)	(0.770 0.773)	(0.771 0.774)
$I_{sd1}\;\left(\mu A\right)$	(0.450 0.773)	(0.591 0.753)	(0.649 0.769)	(0.625 0.836)	(0.719 0.831)	(0.727 0.760)	(0.726 0.752)
$I_{sd2}\;\left(\mu A\right)$	(0.105 0.453)	(0.125 0.336)	(0.122 0.252)	(0.175 0.272	(0.197 0.252)	(0.210 0.241)	(0.208 0.229)
$R_{s}\;\left(\Omega \right)$	(0.045 0.622)	(0.035 0.36)	(0.023 0.63)	(0.025 0.063)	(0.026 0.053)	(0.034 0.042)	(0.028 0.035)
$R_{sh}\;\left(\Omega \right)$	(40.25 55.43)	(38.81 54.36)	(48.56 57.62)	(49.53 59.87)	(49.43 58.43)	(55.42 55.53)	(55.39 55.45)
$n_{1}$	(1.104 1.844)	(1.192 1.579)	(1.003 1.685)	(1.013 1.492)	(1.005 1.647)	(1.023 1.361)	(1.019 1.251)
$n_{2}$	(1.034 1.645)	(1.031 1.643)	(1.323 1.832)	(1.226 1.771)	(1.026 1.642)	(1.234 1.446)	(1.128 1.365)

**Table 14:** Optimal parameters with various irradiance under 25 °C on KC200GT.

Parameters	Irradiance				
	$200\;W/m^{2}$	$400\;W/m^{2}$	$600\;W/m^{2}$	$800\;W/m^{2}$	$1000\;W/m^{2}$
SDM					
$I_{Ph}\;\left(A\right)$	1.646154479	3.28784893	4.934307945	6.571327377	8.216891461
$I_{sd}\;\left(\mu A\right)$	5.20997E-10	1.48987E-09	3.86144E-09	9.53063E-10	2.24195E-09
$R_{s}\;\left(\Omega \right)$	0.381111453	0.35357862	0.33733826	0.357335312	0.343814047
$R_{sh}\;\left(\Omega \right)$	690.1466111	752.0893857	743.0015919	743.523157	763.5351733
*n*	1.003243464	1.05503875	1.104020542	1.035319708	1.076410285
RMSE	0.001418466	0.0014262	0.001297668	0.00163097	0.001539031
DDM					
$I_{Ph}\;\left(A\right)$	1.645937346	3.287848931	4.934307944	6.570513347	8.21684217
$I_{sd1}\;\left(\mu A\right)$	4.84562E-10	1.48987E-09	1.51823E-17	5.52985E-08	2.19172E-06
$I_{sd2}\;\left(\mu A\right)$	1.53073E-05	1.12133E-18	3.86144E-09	5.03735E-10	2.23103E-09
$R_{S}\;\left(\Omega \right)$	0.383131626	0.353578609	0.33733826	0.361106414	0.343861097
$R_{sh}\;\left(\Omega \right)$	709.501561	752.089397	743.0016353	800.6518791	767.6196281
$n_{1}$	1	1.05503876	3.900635458	1.525357331	3.999999999
$n_{2}$	4	3.999398783	1.104020542	1.008760998	1.076176379
RMSE	0.001409086	0.0014262	0.001297668	0.001504603	0.001538902

**Table 15:** Optimal parameters with various irradiance under 25 °C on SM55.

Parameters	Irradiance				
	$200\;W/m^{2}$	$400\;W/m^{2}$	$600\;W/m^{2}$	$800\;W/m^{2}$	$1000\;W/m^{2}$
SDM					
	0.691248291	1.381057893	2.067765031	2.759093824	3.436047438
	2.71212E-07	4.43297E-07	2.03018E-07	3.13152E-07	6.45107E-07
	0.082108349	0.203278773	0.310907452	0.308145026	0.277201933
	452.878803	493.3582679	507.4557269	570.6471712	2680.813018
	1.436656611	1.483204363	1.410423218	1.44849796	1.514343268
RMSE	0.000692735	0.002057132	0.002124264	0.004265301	0.00771571
DDM					
	0.691957957	1.380954183	2.059183231	2.751027153	3.445209297
	3.61469E-06	1.49094E-06	2.24877E-05	1.17431E-06	5.9976E-07
	1.63577E-07	3.88515E-07	8.98011E-07	9.86533E-11	8.92042E-05
	0.229008693	0.189036405	0.205070277	0.210430191	0.282575756
	435.9639476	492.4349288	1716.653153	1399.596578	2427.107852
	3.659775367	2.418359716	3.956451963	1.575018295	1.509427466
	1.390555753	1.470279235	1.551328204	2.241096258	3.574506931
RMSE	0.000626035	0.002039828	0.005757756	0.006267057	0.00970765

**Table 16:** Optimal parameters with various irradiance under 25 °C on ST40.

Parameters	Irradiance				
	$200\;W/m^{2}$	$400\;W/m^{2}$	$600\;W/m^{2}$	$800\;W/m^{2}$	$1000\;W/m^{2}$
SDM					
$I_{Ph}\;\left(A\right)$	0.531425096	1.064508113	1.597308387	2.124347536	2.665647952
$I_{sd}\;\left(\mu A\right)$	2.94879E-06	6.99215E-06	5.44988E-06	5.81026E-06	6.9704E-06
$R_{s}\;\left(\Omega \right)$	0.820589566	0.789156411	0.945182902	0.975217874	0.999161627
$R_{sh}\;\left(\Omega \right)$	370.9477282	445.5980471	548.2669693	855.4603519	826.6014302
*n*	1.849813542	1.974441249	1.926475111	1.931219317	1.953940203
RMSE	0.000721193	0.002693291	0.003248761	0.00604703	0.00605464
DDM					
$I_{Ph}\;\left(A\right)$	0.532396553	1.060465515	1.595062911	2.130825324	2.662431127
$I_{sd1}\;\left(\mu A\right)$	3.2077E-06	3.72141E-06	6.15112E-06	2.52968E-07	2.106E-06
$I_{sd2}\;\left(\mu A\right)$	1.96347E-07	5.53546E-05	6.30906E-06	5.81131E-06	6.31659E-06
$R_{S}\;\left(\Omega \right)$	0.740251312	0.891233534	0.89357389	0.971229541	1.002377667
$R_{sh}\;\left(\Omega \right)$	354.6031776	564.3816878	557.9050094	567.9134574	980.0384572
$n_{1}$	1.863751702	1.879419022	1.945194078	2.436590166	2.879340474
$n_{2}$	2.504584035	3.603596456	3.72074148	1.932546679	1.939396996
RMSE	0.000766834	0.003200033	0.004157005	0.005530575	0.005728042

**Table 17:** Optimal parameters with various temperatures under 1000 irradiance on KC200GT.

Parameters	Temperature		
	25 °C	50 °C	75 °C
SDM			
$I_{Ph}\;\left(A\right)$	8.216891462	8.295305202	8.37766296
$I_{sd}\;\left(\mu A\right)$	2.24195E-09	1.25953E-07	1.63082E-06
$R_{s}\;\left(\Omega \right)$	0.343814049	0.335654705	0.342497962
$R_{sh}\;\left(\Omega \right)$	763.53516	953.8900519	790.5583357
*n*	1.076410281	1.117292259	1.101479934
RMSE	0.001539031	0.002746513	0.00447293
DDM			
$I_{Ph}\;\left(A\right)$	8.21684217	8.295305203	8.377662961
$I_{sd1}\;\left(\mu A\right)$	2.19168E-06	1.25953E-07	1.63082E-06
$I_{sd2}\;\left(\mu A\right)$	2.23103E-09	6.55032E-19	1.84724E-16
$R_{S}\;\left(\Omega \right)$	0.343861095	0.335654704	0.342497962
$R_{sh}\;\left(\Omega \right)$	767.619613	953.8899695	790.5582688
$n_{1}$	3.999999998	1.11729226	1.101479936
$n_{2}$	1.076176385	2.337556414	3.435675905
RMSE	0.001538902	0.002746513	0.00447293

**Table 18:** Optimal parameters with various temperatures under 1000 irradiance on SM55.

Parameters	Temperature		
	25 °C	40 °C	60 °C
SDM			
$I_{Ph}\;\left(A\right)$	8.216891462	8.295305202	8.37766296
$I_{sd}\;\left(\mu A\right)$	2.24195E-09	1.25953E-07	1.63082E-06
$R_{s}\;\left(\Omega \right)$	0.343814049	0.335654705	0.342497962
$R_{sh}\;\left(\Omega \right)$	763.53516	953.8900519	790.5583357
*n*	1.076410281	1.117292259	1.101479934
RMSE	0.001539031	0.002746513	0.00447293
DDM			
$I_{Ph}\;\left(A\right)$	8.21684217	8.295305203	8.377662961
$I_{sd1}\;\left(\mu A\right)$	2.19168E-06	1.25953E-07	1.63082E-06
$I_{sd2}\;\left(\mu A\right)$	2.23103E-09	6.55032E-19	1.84724E-16
$R_{S}\;\left(\Omega \right)$	0.343861095	0.335654704	0.342497962
$R_{sh}\;\left(\Omega \right)$	767.619613	953.8899695	790.5582688
$n_{1}$	3.999999998	1.11729226	1.101479936
$n_{2}$	1.076176385	2.337556414	3.435675905
RMSE	0.001538902	0.002746513	0.00447293

**Table 19:** Optimal parameters with various temperatures under 1000 irradiance on ST40.

Parameters	Temperature			
	25 °C	40 °C	50 °C	70 °C
SDM				
$I_{Ph}\;\left(A\right)$	2.662688142	2.676833598	2.686835757	2.692277091
$I_{sd}\;\left(\mu A\right)$	5.31093E-06	9.71415E-06	2.12399E-05	8.6798E-05
$R_{s}\;\left(\Omega \right)$	1.018966041	1.098934605	1.146647832	1.126380689
$R_{sh}\;\left(\Omega \right)$	749.7105673	466.6516864	358.8266461	366.6982882
*n*	1.914374291	1.796421115	1.736300707	1.725939139
RMSE	0.005689369	0.004236714	0.002476438	0.000786502
DDM				
$I_{Ph}\;\left(A\right)$	2.664663439	2.674274118	2.682205432	2.692794624
$I_{sd1}\;\left(\mu A\right)$	4.91026E-06	9.20722E-06	1.10215E-05	2.12155E-06
$I_{sd2}\;\left(\mu A\right)$	3.92577E-05	4.2153E-06	3.49233E-05	8.54212E-05
$R_{S}\;\left(\Omega \right)$	1.000701581	1.081914243	1.10036569	1.127837821
$R_{sh}\;\left(\Omega \right)$	847.5917973	581.3433446	500.7198612	358.2237529
$n_{1}$	1.906321607	1.791423861	3.833798357	3.35289197
$n_{2}$	3.282423889	2.310094454	1.811753621	1.723358005
RMSE	0.006145747	0.003621486	0.003524141	0.000788367

## Data Availability

Not applicable.

## Nomenclature

CPA	Shuffled frog leading algorithm	$N_{S}$	Number of series solar cells
MLCPA	Multi-strategy learning boosted colony predation algorithm	$X_{b}$	Global best position
SDM	Single diode model	$f_{\mathrm{value}\;}$	Global best solution
DDM	Double diode model	ub	Upper boundary
PV	Photovoltaic	lb	Lower boundary
RMSE	Root mean square error	*M*	Maximum iterations
RTC France	Name of solar cell	*N*	Population size
Photowatt-PWP 201	Name of photovoltaic module	*S*	The strength of prey
IAE	Absolute error	*D*	Distance of individual and prey
RE	Relative error	*a*	Control parameter
SM55	Name of mono-crystalline PV module	*t*	Iterative generation
ST40	Name of thin-film PV module	$R_{S}$	Series resistor
KC200GT	Multicrystal photovoltaic module	$R_{sh}$	Parallel resistor
$I_{L}$	Output current	$n_{1},n_{2},n_{3}$	Diode ideality factor
$I_{ph}$	Photo-generated current	$V_{L}$	Output voltage
$I_{d},I_{d1},I_{d2}$	Diode current	*T*	Kelvin temperature
$I_{Sh}$	Shunt resistor current	*K*	Boltzmann constant
$I_{sd},I_{sd1},I_{sd2}$	Reverse saturated current	$N_{P}$	Number of parallel solar cells
